# Galectin-3 for prediction of cardiac function compared to NT-proBNP in individuals with prediabetes and type 2 diabetes mellitus

**DOI:** 10.1038/s41598-021-98227-x

**Published:** 2021-09-24

**Authors:** Volker H. Schmitt, Jürgen H. Prochaska, Annegret S. Föll, Andreas Schulz, Karsten Keller, Omar Hahad, Thomas Koeck, Sven-Oliver Tröbs, Steffen Rapp, Manfred Beutel, Norbert Pfeiffer, Konstantin Strauch, Karl J. Lackner, Thomas Münzel, Philipp S. Wild

**Affiliations:** 1 Department of Cardiology, Cardiology I, University Medical Center, Johannes Gutenberg University Mainz, Langenbeckstr. 1, 55131 Mainz, Germany; 2 German Center for Cardiovascular Research (DZHK), Partner Site Rhine-Main, Mainz, Germany; 3 Preventive Cardiology and Preventive Medicine, Department of Cardiology, University Medical Center, Johannes Gutenberg University Mainz, Langenbeckstr. 1, 55131 Mainz, Germany; 4 Center for Thrombosis and Hemostasis (CTH), University Medical Center, Johannes Gutenberg University Mainz, Langenbeckstr. 1, 55131 Mainz, Germany; 5 Medical Clinic VII, Department of Sports Medicine, University Hospital Heidelberg, Im Neuenheimer Feld 410, 69120 Heidelberg, Germany; 6 Department of Psychosomatic Medicine and Psychotherapy, University Medical Center, Johannes Gutenberg University Mainz, Langenbeckstr. 1, 55131 Mainz, Germany; 7 Department of Ophthalmology, University Medical Center, Johannes Gutenberg University Mainz, Langenbeckstr. 1, 55131 Mainz, Germany; 8 Institute of Medical Biostatistics, Epidemiology and Informatics (IMBEI), University Medical Center, Johannes Gutenberg University Mainz, Obere Zahlbacher Str. 69, 55131 Mainz, Germany; 9 Institute of Clinical Chemistry and Laboratory Medicine, University Medical Center, Johannes Gutenberg University Mainz, Langenbeckstr. 1, 55131 Mainz, Germany

**Keywords:** Biomarkers, Cardiology, Endocrinology

## Abstract

Use of galectin-3 for assessing cardiac function in prediabetes and type 2 diabetes mellitus (T2DM) needs to be established. Within the Gutenberg Health Study cohort (N = 15,010, 35–74 years) patient characteristics were investigated regarding galectin-3 levels. Prognostic value of galectin-3 compared to NT-proBNP concerning cardiac function and mortality was assessed in individuals with euglycaemia, prediabetes and T2DM in 5 years follow-up. Higher galectin-3 levels related to older age, female sex and higher prevalence for prediabetes, T2DM, cardiovascular risk factors and comorbidities. Galectin-3 cross-sectionally was related to impaired systolic (β − 0.36, 95% CI − 0.63/− 0.09; P = 0.008) and diastolic function (β 0.014, 95% CI 0.001/0.03; P = 0.031) in T2DM and reduced systolic function in prediabetes (β − 0.34, 95% CI − 0.53/− 0.15; P = 0.00045). Galectin-3 prospectively related to systolic (β − 0.656, 95% CI − 1.07/− 0.24; P = 0.0021) and diastolic dysfunction (β 0.0179, 95% CI 0.0001/0.036; P = 0.049), cardiovascular (hazard ratio per standard deviation of galectin-3 (HR_perSD_) 1.60, 95% CI 1.39–1.85; P < 0.0001) and all-cause mortality (HR_perSD_ 1.36, 95% CI 1.25–1.47; P < 0.0001) in T2DM. No relationship between galectin-3 and cardiac function was found in euglycaemia, whereas NT-proBNP consistently related to reduced cardiac function. Prospective value of NT-proBNP on cardiovascular and all-cause mortality was higher. NT-proBNP was superior to galectin-3 to assess reduced systolic and diastolic function.

## Introduction

Type 2 diabetes mellitus (T2DM) represents an epidemic metabolic disease with increasing prevalence affecting over 463 million people today. It has been estimated that approximately 700 million individuals would suffer from T2DM in the year 2045^1^. Underlying reasons for this marked increase are the modified living conditions with sedentary lifestyle and less physical activity resulting in an increase of obesity in a rising world population with longevity^2^. Since T2DM increases the risk for various ailments and diseases such as cancer and cardiovascular diseases (CVD), life expectancy and quality of life of affected individuals are considerably reduced^3–5^. Due to microvascular and macrovascular alterations, the risk for CVD is twice as high in patients with T2DM compared to individuals with euglycaemia. Approximately 80% of T2DM-associated deaths are related to a progress in CVD and occurrence of their acute manifestations including heart failure, myocardial infarction and stroke^6–8^. An elevated risk for CVD is not only present in T2DM but also in the early precursor form prediabetes^4,9^.

Impaired glucose metabolism was causative for alterations in the myocardium in combination with other cardiovascular risk factors (CVRF), but also singularly in absence of further CVRF and CVD such as arterial hypertension and/or coronary artery disease. Impaired glucose metabolism results in higher ventricle stiffness and reduced cardiac compliance followed by ventricular dysfunction^10^. Pathophysiologically, the hyperglycaemia triggered formation of advanced glycation end products and reactive oxygen species were found to be responsible mechanisms of cardiac impairment^11^ accompanied by altered cardiac metabolism and cardiac fibrosis contributing to an elevated risk for development of heart failure^8,12^.

In diagnostics of heart failure, NT-proBNP represents the gold-standard laboratory parameter, but also galectin-3 revealed promising results regarding an association with the presence of cardiac dysfunction^13,14^. Since galectin-3 was demonstrated to be involved in inflammatory and fibrotic processes as well as cardiac remodeling^15,16^, we hypothesized that galectin-3 might be a specific and complementary biomarker particularly for hyperglycaemia induced cardiac impairment. Aim of the study was to assess galectin-3 as a biomarker for cardiac function in people with euglycaemia, prediabetes and type 2 diabetes mellitus. By this, the association of galectin-3 to left ventricular systolic and diastolic function was investigated in regard to diabetic state in a large population-based study cohort. The findings were confronted with NT-proBNP in order to refer the results to an established biomarker and gold-standard in diagnosis of heart failure.

## Materials and methods

### Design of the study

The Gutenberg Health Study (GHS) represents a large prospective, observational, population-based study in Mid-Western Germany including 15,010 individuals aged 35–74 years starting in 2007 after approval by the data protection officer and the Ethics Committee of the State Chamber of Physicians of Rhineland-Palatinate, Germany (reference no.: 837.020.07[5555], date of approval: 22.03.2007). All individuals provided written informed consent before study enrolment and the study procedures are conducted in line with the principles outlined in the Declaration of Helsinki and the recommendations for Good Epidemiological Practice. The rationale and design of the GHS were published previously^17,18^. Data acquisition was performed using a highly standardized investigational protocol at the study platform. Data including inter alia anthropometrics, CVRF (age, sex, arterial hypertension, diabetes mellitus, dyslipidaemia, obesity, smoking, positive family history for myocardial infarction and/or stroke), clinical comorbidities including CVD (coronary artery disease, myocardial infarction, stroke or transient ischemic attack, heart failure, atrial fibrillation, peripheral artery disease), medication intake (based on the Anatomical Therapeutic Chemical (ATC) classification code of medication provided by the World Health Organization), and venous blood sample was collected. Standardized echocardiographic examination (echocardiography machine iE33, Philips, Hamburg, Germany and ultrasonic probe S5-1, Royal Philips Electronics, Amsterdam, the Netherlands) was conducted by trained medical-technical assistants in left lateral position. Ejection fraction (EF) was assessed with the Simpson method. E and E′ were acquired via pulsed wave doppler and tissue doppler imaging of the lateral mitral annulus in four chamber view. The recorded images were analysed by specialized physicians. HbA1c concentration was assessed with standardized high-performance liquid chromatography assays (Bio-Rad Laboratories, Hercules, California, USA and Abbott Laboratories, Abbott Park, Illinois, USA), Galectin-3 was measured via enzyme-linked immunosorbent assay (BG Medicine, Waltham, Massachusetts, USA) and NT-proBNP was assessed with an immunoassay in sandwich procedure (Roche Diagnostics, Mannheim, Germany). Of every individual data was recollected using the same investigational protocol including echocardiographic assessment and blood sampling at the study platform after 5 years for follow-up investigation.

### Definition of prediabetes and type 2 diabetes mellitus

Euglycaemia, prediabetes and T2DM were defined according to the current recommendations of the American Diabetes Association^19^. Hence, euglycaemia was present with HbA1c levels < 5.7% (< 39 mmol/mol). Prediabetes was defined as HbA1c levels between 5.7 and 6.4% (39–47 mmol/mol). Individuals were categorized in the T2DM group if T2DM was diagnosed by a physician and/or intake of antidiabetic drugs and/or HbA1c levels ≥ 6.5% (≥ 48 mmol/mol). Individuals with other forms of diabetes mellitus than T2DM (e.g. type 1 diabetes mellitus, gestational diabetes mellitus, diabetes mellitus following pancreatitis) as well as subjects with euglycaemic HbA1c levels and fasting glucose > 125 mg/dl were excluded from the present study.

### Statistical analyses

Categorical variables were given by relative and absolute frequencies, continuous variables were described by mean and standard deviation (SD) or for skewed distribution (defined as│skewness│ > 1) by median and interquartile range. For statistical comparison of categorical data Fisher’s exact or chi-squared tests were performed. Mann–Whitney U test or Student t-test were used for continuous traits. In order to assess the relation of systolic and diastolic cardiac function as well as levels of NT-proBNP and galectin-3 in different glucose states, multiple linear regression models were performed with baseline data and after 5 years follow-up. Individuals with impaired systolic or diastolic function at baseline were excluded from follow-up analysis. Cross-sectional and prospective association between galectin-3 or NT-proBNP and cardiac function was performed using three multiple regression models: model 1 adjusted for sex and age, model 2 additionally adjusted for CVRF (except diabetes mellitus) and model 3 in addition adjusted for comorbidities and intake of heart failure medication (ATC codes C09 for angiotensin converting enzyme inhibitors, C07 for beta blocking agents, C03 for diuretics, C01AA for digitalis glycosides, C01DA for organic nitrates including C01DA08 for isosorbide dinitrate) as potential confounders for the relation of diabetic phenotype with impaired cardiac function. To investigate the association between mortality and galectin-3 as well as NT-proBNP in prediabetes and T2DM compared to euglycaemia, Cox regression models and Kaplan–Meier curves were computed (model 1: adjustment for sex and age; model 2: additional adjustment for CVRF excluding diabetes mellitus; model 3: additional adjustment for systolic (EF) and diastolic (log(E/E′) function). P values < 0.05 were considered as significant association. Statistical analyses were performed with the software package R (R Core Team, R version 4.0.5 (2021-03-31). R: a language and environment for statistical computing. R Foundation for Statistical Computing, Vienna, Austria, 2021, URL https://www.R-project.org/).

## Results

### Prevalence of diabetic phenotypes and comorbidities related to galectin-3 levels

**Figure 1:**
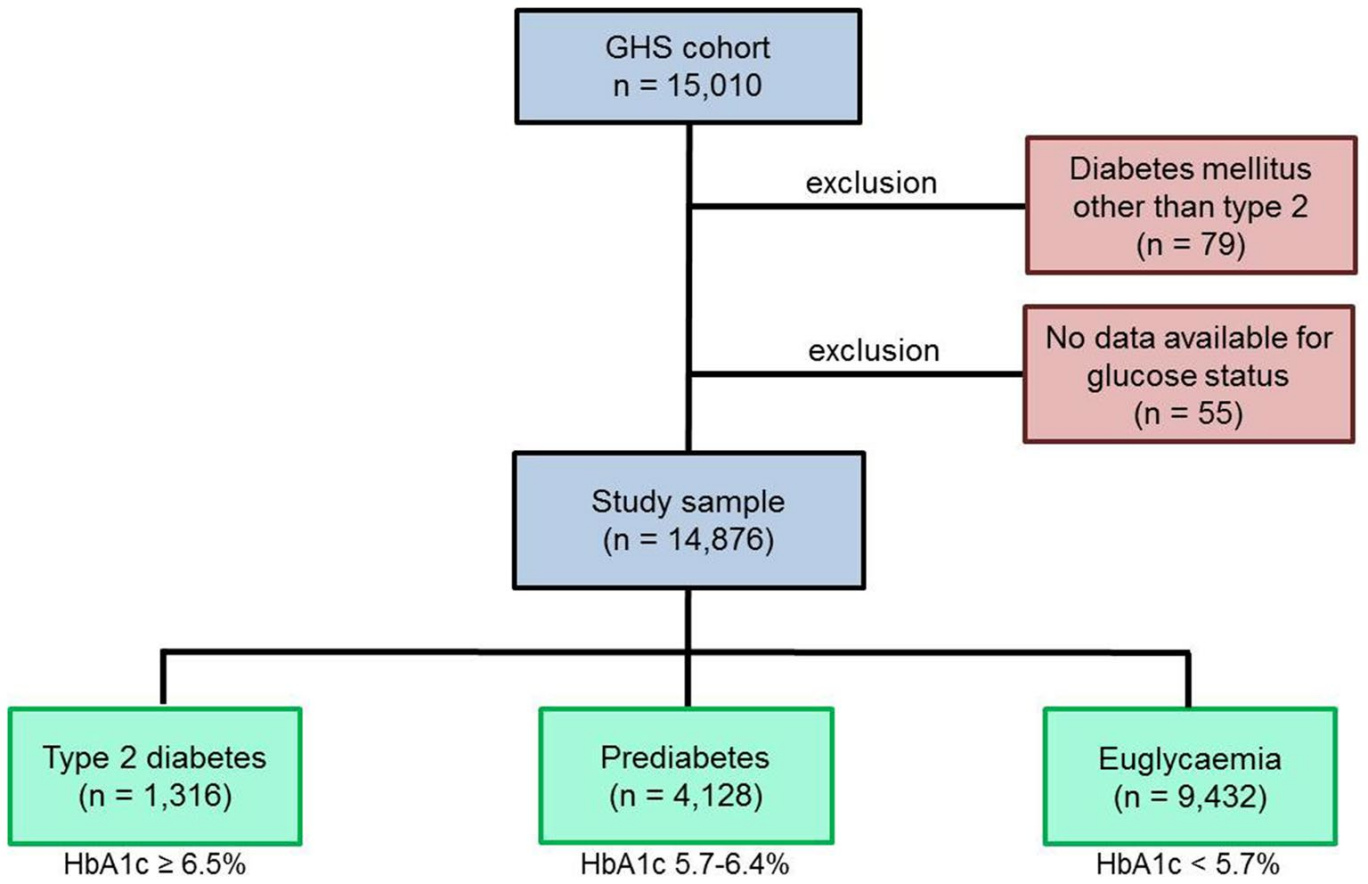
Study sample. Euglycaemia was defined as HbA1c < 5.7%, no T2DM diagnosis by a physician and no intake of diabetic medication. Prediabetes was declared as HbA1c 5.7–6.4%, no T2DM diagnosis by a physician and no intake of diabetic medication. T2DM was present if HbA1c ≥ 6.5 or diagnosis of T2DM by a physician or intake of diabetic medication. Individuals without available data for glucose status and all diabetes types other than T2DM were excluded. In total, 14.876 individuals were included into the study. The blue boxes show the GHS study cohort of which the individuals were included into the present study. The red boxes illustrate excluded participants. In the green boxes the subgroups of the final study sample are displayed. GHS: Gutenberg Health Study.

In total 14,892 participants were included into the present study (Fig. 1). The SD of galectin-3 was 5 ng/ml. The first tertile (galectin-3 ≤ 12.2 ng/ml) included 4925 people, the second tertile (galectin-3 > 12.2 to ≤ 15.3 ng/ml) comprised 4934 subjects and the tird tertile (galectin-3 > 15.3 ng/ml) involved 4924 individuals. Lower galectin-3 levels were associated with euglycaemic state, whereas higher galectin-3 was related to a higher prevalence of prediabetes (increase of approximately 50% from the first to the third tertile) and T2DM (almost tripled prevalence from the first to the third tertile) (Supplemental Fig. 1). With raising galectin-3 levels individuals were older and more often female, and also higher prevalence of arterial hypertension, dyslipidaemia, obesity and all assessed comorbidities was present. As expected, higher galectin-3 levels were related to higher prevalence of heart failure. Baseline characteristics of the study cohort are provided in Table 1.

**Table 1:** Galectin-3 tertiles and the prevalence of diabetic status, cardiovascular risk factors, comorbidities, cardiac function (n = 14,876).

	1st tertile of Galectin-3 (≤ 12.2 ng/ml, n = 4925)	2nd tertile of Galectin-3 (> 12.2 to ≤ 15.3 ng/ml, n = 4934)	3rd tertile of Galectin-3 (> 15.3 ng/ml, n = 4924)
Age [years], mean (SD)	51.2 (10.4)	54.8 (10.9)	59.0 (10.6)
Female sex, % (n)	45.5 (2240)	49.3 (2431)	53.0 (2609)
**Glucose state**			
Euglycaemia, % (n)	73.1 (3602)	64.0 (3159)	53.2 (2622)
Prediabetes, % (n)	21.8 (1076)	28.3 (1394)	33.0 (1626)
Type 2 diabetes mellitus, % (n)	5.0 (247)	7.7 (381)	13.7 (676)
**Traditional cardiovascular risk factors other than diabetes**			
Arterial hypertension, % (n)	39.3 (1935)	48.6 (2398)	61.4 (3018)
Dyslipidaemia, % (n)	34.7 (1707)	44.0 (2170)	54.2 (2669)
Family history of myocardial infarction/stroke, % (n)	21.7 (1069)	21.9 (1080)	22.6 (1115)
Obesity, % (n)	17.1 (841)	23.8 (1175)	34.6 (1704)
Smoking, % (n)	19.9 (979)	20.1 (991)	18.4 (904)
**Comorbidities**			
Atrial fibrillation, % (n)	1.6 (78)	2.1 (104)	4.5 (222)
Cancer, % (n)	7.6 (713)	10.9 (448)	13.9 (182)
Chronic kidney disease, % (n)	0.6 (31)	0.8 (40)	1.7 (84)
Congestive heart failure, % (n)	0.5 (24)	1.2 (57)	2.3 (115)
HFpEF, % (n)	1.6 (76)	3.8 (186)	6.6 (313)
HFrEF, % (n)	0.6 (31)	0.9 (42)	2.0 (97)
Coronary artery disease, % (n)	2.2 (110)	4.0 (199)	6.5 (319)
Myocardial infarction, % (n)	1.2 (59)	2.6 (127)	5.0 (248)
Peripheral artery disease, % (n)	2.5 (124)	2.5 (124)	4.9 (242)
Stroke, % (n)	0.9 (42)	1.7 (82)	2.9 (145)
Venous thromboembolism, % (n)	2.6 (127)	4.3 (211)	5.0 (244)
**Medication**			
Alpha glucosidase inhibitors	0.1 (5)	0.0 (2)	0.1 (4)
Biguanides	1.6 (76)	3.6 (174)	6.2 (304)
Dipeptidyl peptidase 4 (DPP-4) inhibitors	0.1 (4)	0.2 (10)	0.3 (16)
Sulfonylureas	0.5 (25)	1.0 (49)	2.0 (98)
Thiazolidinediones	0.1 (6)	0.2 (12)	0.3 (17)
Combinations of oral blood glucose lowering drugs	0.3 (15)	0.2 (10)	0.6 (30)
Insulin and insulin analogues	0.9 (43)	1.5 (72)	3.1 (151)
Beta blocking agents	9.7 (469)	15.7 (765)	25.6 (1253)
Angiotensin-converting enzyme (ACE) inhibitors	8.8 (424)	15.1 (734)	23.2 (1135)
Angiotensin II receptor blockers (ARB)	6.7 (321)	9.9 (484)	15.2 (743)
**Biomarkers**			
CRP [mg/l], median (Q1/Q3)	1.30 (0.50/2.40)	1.50 (0.53/3.00)	2.10 (1.10/4.30)
Galectin-3 [ng/ml], median (Q1/Q3)	10.50 (9.34/11.38)	13.65 (12.94/14.42)	17.69 (16.30/20.00)
NT-proBNP [pg/ml], median (Q1/Q3)	50.08 (25.40/91.76)	58.31 (30.26/109.83)	75.36 (37.52/151.23)
**Cardiac function**			
Left ventricular ejection fraction [%], median (Q1/Q3)	63.4 (60.0/67.0)	63.5 (60.0/67.1)	63.4 (59.7/67.1)
Left ventricular E/E′, median (Q1/Q3)	6.72 (5.63/8.20)	7.18 (5.88/8.92)	7.80 (6.34/9.71)
Heart failure medication, % (n)	19.9 (965)	30.8 (1506)	45.5 (2227)

Continuous variables are expressed by mean values with standard deviation (SD) or median with interquartile range (Q1/Q3). Discrete variables are described through relative and absolute frequencies. Heart failure medication according to ATC codes C09 (angiotensin converting enzyme inhibitors), C07 (beta blocking agents), C03 (diuretics), C01AA (digitalis glycosides), C01DA (organic nitrates) including C01DA08 (isosorbide dinitrate).

*ATC code* Anatomical Therapeutic Chemical classification code of medication, *CRP* C-reactive protein, *EF* ejection fraction, *eGFR* estimated glomerular filtration rate, *HFpEF* heart failure with preserves ejection fraction, *HFrEF* heart failure with restricted ejection fraction, *BNP* brain natriuretic peptide.

### Galectin-3 and cardiac function in prediabetes and type 2 diabetes mellitus

The associations between galectin-3 level and systolic and diastolic function in prediabetes and T2DM were assessed with multiple linear regression models (Table 2). Diastolic or systolic dysfunction was not associated to galectin-3 in euglycaemic individuals. In prediabetes and T2DM, systolic function was reduced with increasing galectin-3 levels independently of age, sex, traditional CVRF, comorbidities and intake of heart failure medication. Diastolic dysfunction (E/E′ ratio) was associated with increasing galectin-3 levels in prediabetes after adjustment for sex and age, but after additional adjustment for traditional CVRF the association was attenuated. Contrary, in T2DM a relevant association between diastolic dysfunction and galectin-3 levels was still observed in the fully adjusted model. Altogether, increasing galectin-3 levels were associated with occurrence of impaired systolic and diastolic cardiac function in T2DM as well as with an impaired systolic cardiac function in prediabetes, whereas no association was found in euglycaemic subjects.

**Table 2:** Cross-sectional association between galectin-3 and cardiac function in prediabetes and type 2 diabetes mellitus.

	Model 1: age, sex		Model 2: add. traditional CVRF		Model 3: add. traditional CVRF, comorbidities, heart failure medication	
	β-estimate (95% CI)	P value	β-estimate (95% CI)	P value	β-estimate (95% CI)	P value
**Systolic function**						
EF in prediabetes vs. euglycaemia	− 0.281 (− 0.505; − 0.0563)	0.014	− 0.192 (− 0.421; 0.0362)	0.099	− 0.136 (− 0.364; 0.0928)	0.24
EF in diabetes vs. euglycaemia	− 0.573 (− 0.945; − 0.200)	0.0026	− 0.385 (− 0.772; 0.00282)	0.052	− 0.197 (− 0.586; 0.193)	0.32
EF ~ Galectin-3 [SD] in euglycaemia	− 0.0484 (− 0.173; 0.0763)	0.45	− 0.0183 (− 0.144; 0.107)	0.78	0.00539 (− 0.121; 0.132)	0.93
EF ~ Galectin-3 [SD] in prediabetes	− 0.423 (− 0.611; − 0.234)	< 0.0001	− 0.397 (− 0.587; − 0.208)	< 0.0001	− 0.340 (− 0.530; − 0.150)	0.00045
EF ~ Galectin-3 [SD] in diabetes	− 0.521 (− 0.787; − 0.255)	0.00012	− 0.509 (− 0.775; − 0.242)	0.00019	− 0.361 (− 0.628; − 0.0941)	0.008
**Diastolic function**						
log (E/E′) in prediabetes vs. euglycaemia	0.0376 (0.0270; 0.0482)	< 0.0001	0.0157 (0.00519; 0.0263)	0.0035	0.0164 (0.00579; 0.0271)	0.0025
log (E/E′) in diabetes vs. euglycaemia	0.127 (0.109; 0.144)	< 0.0001	0.0677 (0.0498; 0.0856)	< 0.0001	0.0613 (0.0431; 0.0794)	< 0.0001
log (E/E′) ~ Galectin-3 [SD] in euglycaemia	0.0141 (0.00825; 0.0200)	< 0.0001	0.00480 (− 0.00101; 0.0106)	0.11	0.00425 (− 0.00164; 0.0101)	0.16
log (E/E′) ~ Galectin-3 [SD] in prediabetes	0.0116 (0.00273; 0.0205)	0.01	− 0.000482 (− 0.00924; 0.00827)	0.91	− 0.00290 (− 0.0118; 0.00595)	0.52
log (E/E′) ~ Galectin-3 [SD] in diabetes	0.0250 (0.0125; 0.0376)	< 0.0001	0.0169 (0.00457; 0.0292)	0.0072	0.0136 (0.00121; 0.0260)	0.031

Multiple linear regression models for investigation of the association between Galectin-3 (increase per standard deviation respectively 5 ng/ml) and EF as well as log(E/E′) in prediabetes and diabetes compared to euglycaemia within the baseline data. Model 1 adjusted for sex and age. Model 2 adjusted for sex, age, hypertension, dyslipidemia, obesity, smoking, FH of MI/stroke. Model 3 adjusted for sex, age, hypertension, dyslipidemia, obesity, smoking, FH of MI/Stroke, atrial fibrillation, chronic kidney disease, chronic liver disease, congestive heart failure, coronary artery disease, myocardial infarction, peripheral artery disease, stroke, venous thromboembolism, heart failure medication intake.

*CVRF* cardiovascular risk factors, *SD* standard deviation.

### Prospective analyses of a possible association between galectin-3 and cardiac function in prediabetes and type 2 diabetes mellitus

To further investigate the link between cardiac function and galectin-3 expression in prediabetes and T2DM, prospective analyses were performed within a 5-years follow-up performing multiple linear regression models (Table 3). Within the 5-years follow-up period, of 4128 people with prediabetes at baseline, follow-up data of 3385 people were available and of these 359 people had diabetes in the follow-up assessment. Of 8035 non-diabetics at baseline, 96 people had diabetes at the follow-up investigation. In individuals with T2DM, increasing galectin-3 levels were associated with reduced systolic and diastolic function after adjustment for age, sex, traditional CVRF, comorbidities and intake of heart failure medication. No significant association between galectin-3 and systolic or diastolic function was found in prediabetic or euglycaemic subjects.

**Table 3:** Prospective association between galectin-3 and cardiac function in prediabetes and type 2 diabetes mellitus.

	Model 1: age, sex		Model 2: add. traditional CVRF		Model 3: add. traditional CVRF, comorbidities, heart failure medication	
	Estimate (95% CI)	P value	Estimate (95% CI)	P value	Estimate (95% CI)	P value
**Systolic function**						
EF in prediabetes vs. euglycaemia	0.134 (− 0.168; 0.436)	0.38	0.337 (0.0306; 0.643)	0.031	0.353 (0.0441; 0.662)	0.025
EF in diabetes vs. euglycaemia	− 1.64 (− 2.14; − 1.14)	< 0.0001	− 1.19 (− 1.71; − 0.677)	< 0.0001	− 1.14 (− 1.66; − 0.616)	< 0.0001
EF ~ Galectin-3 [SD] in euglycaemia	0.159 (− 0.000104; 0.318)	0.05	0.214 (0.0545; 0.374)	0.0086	0.227 (0.0653; 0.389)	0.0059
EF ~ Galectin-3 [SD] in prediabetes	− 0.0747 (− 0.334; 0.184)	0.57	− 0.00238 (− 0.262; 0.257)	0.99	0.00790 (− 0.254; 0.270)	0.95
EF ~ Galectin-3 [SD] in T2DM	− 0.767 (− 1.18; − 0.352)	0.00029	− 0.782 (− 1.20; − 0.367)	0.00023	− 0.656 (− 1.07; − 0.239)	0.0021
**Diastolic function**						
log (E/E′) in prediabetes vs. euglycaemia	0.0206 (0.00771; 0.0335)	0.0017	0.0111 (− 0.00196; 0.0241)	0.096	0.0119 (− 0.00128; 0.0251)	0.077
log (E/E′) in diabetes vs. euglycaemia	0.0584 (0.0369; 0.0798)	< 0.0001	0.0346 (0.0125; 0.0566)	0.0021	0.0350 (0.0127; 0.0574)	0.0021
log (E/E′) ~ Galectin-3 [SD] in euglycaemia	0.00358 (− 0.00323; 0.0104)	0.30	0.000185 (− 0.00663; 0.007)	0.96	− 0.000372 (− 0.0073; 0.00656)	0.92
log (E/E′) ~ Galectin-3 [SD] in prediabetes	− 0.00519 (− 0.0162; 0.00583)	0.36	− 0.00917 (− 0.0202; 0.00184)	0.1	− 0.00992 (− 0.0211; 0.00125)	0.082
log (E/E′) ~ Galectin-3 [SD] in T2DM	0.0175 (− 0.000141; 0.0352)	0.052	0.0188 (0.00117; 0.0364)	0.037	0.0179 (0.0000921; 0.0357)	0.049

Prospective analyses using multiple linear regression models for the assessment of the association between Galectin-3 (increase per standard deviation respectively 5 ng/ml) and EF as well as log(E/E′) after 5 years in prediabetes and diabetes compared to euglycaemia. Individuals with impaired systolic or diastolic function were excluded from the follow-up investigation. Model 1 adjusted for sex and age. Model 2 adjusted for sex, age, hypertension, dyslipidaemia, obesity, smoking, FH of MI/Stroke. Model 3 adjusted for sex, age, hypertension, dyslipidaemia, obesity, smoking, FH of MI/stroke, atrial fibrillation, chronic kidney disease, chronic liver disease, congestive heart failure, coronary artery disease, myocardial infarction, peripheral artery disease, stroke, venous thromboembolism, heart failure medication intake.

*CVRF* cardiovascular risk factors, *SD* standard deviation, *T2DM* type 2 diabetes.

### Galectin-3 and mortality in prediabetes and type 2 diabetes mellitus

The role of galectin-3 as a marker for mortality in prediabetes and T2DM was investigated with Cox regression models (Table 4). In people with T2DM, cardiovascular death was elevated by 60% and all-cause mortality by 36% per SD increase of galectin-3 after adjustment for age, sex, traditional CVRF and systolic (EF) as well as diastolic function (log(E/E′ ratio)). After adjustment for age, sex, traditional CVRF, cancer and eGFR the risk for cardiovascular mortality was elevated by 53% per SD increase of galectin-3 and all-cause mortality was 40% higher in people with T2DM. In euglycaemia, all-cause mortality was increased by 17% after adjustment for age, sex, traditional CVRF, EF and log(E/E′ ratio) and by 20% per SD increase of galectin-3 after adjustment for age, sex, traditional CVRF, cancer and eGFR. No association was found for people with euglycaemia regarding cardiovascular mortality. In prediabetes no significant association between elevated galectin-3 levels and cardiovascular or all-cause death was seen.

**Table 4:** Association between galectin-3 and cardiovascular as well as all-cause mortality in prediabetes and type 2 diabetes mellitus.

	Model 1: age, sex		Model 2: age, sex, traditional CVRF		Model 3: age, sex, traditional CVRF, EF, log(E/E′)		Model 4: age, sex, traditional CVRF, cancer, eGFR	
	Hazard ratio (95% CI)	P value	Hazard ratio (95% CI)	P value	Hazard ratio (95% CI)	P value	Hazard ratio (95% CI)	P value
**All-cause mortality**								
Galectin-3 [SD] in euglycaemia	1.201 (1.110; 1.300)	< 0.0001	1.190 (1.094; 1.293)	< 0.0001	1.172 (1.077; 1.275)	0.0002	1.204 (1.105; 1.313)	< 0.0001
Galectin-3 [SD] in prediabetes	1.115 (1.006; 1.236)	< 0.039	1.082 (0.972; 1.204)	< 0.15	1.066 (0.958; 1.187)	0.24	1.103 (0.988; 1.232)	0.081
Galectin-3 [SD] in T2DM	1.413 (1.315; 1.518)	< 0.0001	1.375 (1.272; 1.486)	< 0.0001	1.357 (1.254; 1.469)	< 0.0001	1.397 (1.278; 1.527)	< 0.0001
**Cardiovascular mortality**								
Galectin-3 [SD] in euglycaemia	1.177 (0.955; 1.450)	0.13	1.180 (0.933; 1.493)	0.17	1.148 (0.902; 1.460)	0.26	1.128 (0.869; 1.465)	0.37
Galectin-3 [SD] in prediabetes	1.079 (0.829; 1.404)	0.57	1.029 (0.775; 1.367)	0.84	1.005 (0.747; 1.352)	0.97	0.992 (0.731; 1.346)	0.96
Galectin-3 [SD] in T2DM	1.705 (1.492; 1.949)	< 0.0001	1.651 (1.425; 1.914)	< 0.0001	1.603 (1.390; 1.848)	< 0.0001	1.539 (1.273; 1.861)	< 0.0001

Cox regression models for investigation of the association between galectin-3 (increase per standard deviation respectively 5 ng/ml) and cardiovascular as well as all-cause mortality in prediabetes and diabetes compared to euglycaemia. Model 1 adjusted for sex and age; n = 14,783; CV mortality events = 108; all-cause mortality events = 837. Model 2 adjusted for sex, age, hypertension, dyslipidaemia, obesity, smoking, FH of MI/stroke. n = 14,733; CV mortality events = 107; all-cause mortality events = 830. Model 3 adjusted for sex, age, hypertension, dyslipidaemia, obesity, smoking, FH of MI/stroke, EF, log(E/E′). n = 14,561; CV mortality events = 106; all-cause mortality events = 817. Model 4 adjusted for sex, age, hypertension, dyslipidaemia, obesity, smoking, FH of MI/stroke, cancer, eGFR. n = 14,721; CV mortality events = 107; all-cause mortality events = 829.

*EF* ejection fraction, *eGFR* estimated glomerular filtration rate, *CVRF* cardiovascular risk factors, *FH of MI/stroke* family history of myocardial infarction or stroke, *SD* standard deviation, *T2DM* type 2 diabetes.

**Figure 2:**
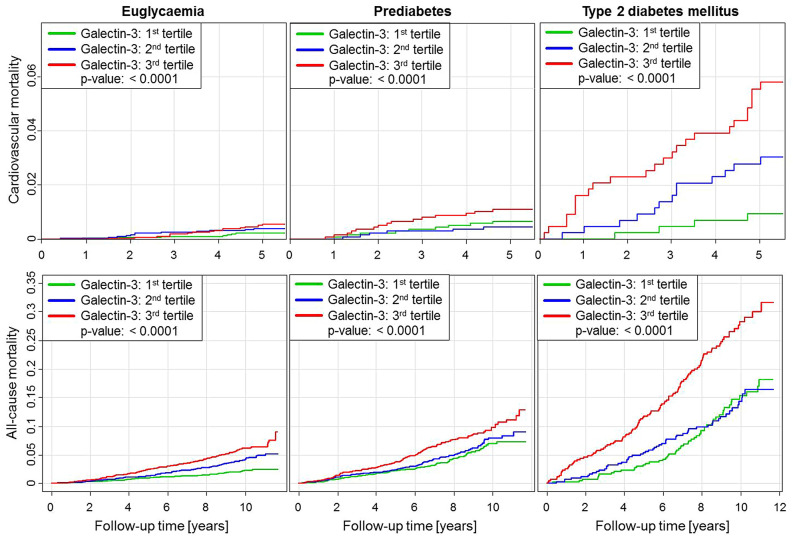
Galectin-3 and mortality in euglycaemia, prediabetes and type 2 diabetes mellitus. Cumulative incidence plots showing the association between Galectin-3 tertiles and cardiovascular as well as all-cause mortality in euglycaemia, prediabetes and type 2 diabetes mellitus. Galectin-3 tertiles are coloured as following: first tertile green, second tertile blue, third tertile red. P for trend is provided in all panels. The figure was created using the software package R (R Core Team, R version 4.0.5 (2021–03-31). R: a language and environment for statistical computing. R Foundation for Statistical Computing, Vienna, Austria, 2021, URL https://www.R-project.org/).

The most distinct relation between galectin-3 level given in tertiles and mortality was seen in T2DM, and in prediabetes the association was still higher compared to euglycaemia (Fig. 2). In euglycaemic and prediabetic individuals the risk for cardiovascular and all-cause mortality was doubled in subjects with high galectin-3 levels (third tertile) compared to lower expression (first tertile). In T2DM, risk for all-cause mortality was comparable in low and intermediate levels but nearly doubled in subjects with high galectin-3 levels (first and second tertile vs. third tertile). In contrast, cardiovascular death was approximately tripled with intermediate levels (first vs. second tertile) and increased to a six-fold risk in high levels of galectin-3 (first vs third tertile). Altogether, the level of galectin-3 significantly correlated with the risk for cardiovascular and all-cause death. Further, galectin-3 revealed a prospective value regarding cardiovascular and all-cause mortality in individuals with T2DM.

### Comparison of galectin-3 and NT-proBNP in prediabetes and type 2 diabetes mellitus

For assessment of validity and diagnostic value of galectin-3 in prediabetes and T2DM in comparison to an established biomarker of heart failure, the results of galectin-3 were compared to NT-proBNP. Higher NT-proBNP levels were associated with impaired systolic and diastolic cardiac function in euglycaemia, prediabetes and T2DM (Supplemental Table 1). This is in contrast to galectin-3, which showed no association to the cardiac function in euglycaemia and was only related to systolic dysfunction in prediabetes. NT-proBNP related to systolic dysfunction in prediabetes and T2DM as well as diastolic dysfunction in euglycaemic individuals (Supplemental Table 2), but no correlation to diastolic dysfunction was found in prediabetes and T2DM. In contrast to NT-proBNP, galectin-3 was associated with systolic and diastolic dysfunction in T2DM, but no relationship was present in euglycaemia and prediabetes. NT-proBNP correlated to cardiovascular and all-cause mortality in euglycaemia, prediabetes and T2DM (Supplemental Table 3). In comparison, galectin-3 correlated with cardiovascular and all-cause mortality in T2DM, and in individuals with euglycaemia elevated galectin-3 levels were associated with an increased risk for death of any cause. Cardiovascular death in T2DM was increased by factor 1.6 per SD of galectin-3 and by factor 2.4 per SD of NT-proBNP. All-cause mortality was increased by 36% per SD of galectin-3 and by 89% per SD of NT-proBNP. In euglycaemia, the risk for all-cause mortality was elevated by 17% per SD of galectin-3 and by 52% per SD of NT-proBNP.

## Discussion

The present study provided a comprehensive analysis of the relationship between galectin-3 and the cardiac function concerning prediabetes and T2DM status compared to euglycaemic individuals in a large population-based study cohort. The results were compared to NT-proBNP as an established biomarker for cardiac function.

The key results of the present study could be summarized as follows: (1) relevant correlations between galectin-3 and both the systolic and the diastolic cardiac function in subjects with T2DM were seen. (2) In prediabetes a relationship between galectin-3 and the systolic function was detected. (3) Galectin-3 also was of prospective value for both systolic and diastolic function after five years of observation in individuals with T2DM. (4) High galectin-3 levels were associated with cardiovascular and all-cause mortality.

Diabetes mellitus is known to have an unfavourable impact on the cardiovascular system leading to heart failure even without further coexisting CVRF^8^. An increase of 1% in HbA1c was demonstrated to elevate the risk for the development of heart failure by 8%^20^. Hyperglycaemia was shown to be accompanied with myocardial fibrosis and inflammation causing reduced myocardial compliance and impairment of the cardiac function^8,10^. Further, T2DM was demonstrated to promote endothelial dysfunction leading to disturbed blood flow and consequently to organ damage including heart failure^21^. Especially the formation of advanced glycation end products and oxidative stress were identified to have a crucial impact in the underlying pathophysiology^22,23^. In this context, not only T2DM but already prediabetes is supposed to elevate the cardiovascular risk^4,24^. In medical attendance of patients with diabetes mellitus, knowledge about the patients’ cardiac state is crucial due to multiple reasons: first, individuals with heart failure and T2DM were shown to more likely have the need for hospitalization compared to euglycaemic heart failure patients^25^. Second, it was shown that in individuals with T2DM mortality was 12-fold elevated in the presence of heart failure compared to T2DM without an impaired cardiac function^26^. Third, within the diagnosis of heart failure the measurement of biomarkers plays a decisive role in daily clinical life to prevent aggravation of disease course.

Besides the already clinically established biomarkers for heart failure like the brain natriuretic peptides, galectin-3 was identified to correlate with a reduced cardiac function^27–29^. This protein was shown to be associated with an echocardiographically dilated and hypertrophied left ventricle, an impaired systolic and diastolic function as well as increased right ventricular pressure^30^. The present study demonstrated a significant association between higher galectin-3 levels and reduced systolic and diastolic cardiac function in subjects with T2DM. Already in the precursor status of prediabetes an association between galectin-3 and the systolic function was detected. Elevated galectin-3 levels in individuals with T2DM were strongly predictive for development systolic and diastolic cardiac impairment at five-years follow-up.

Furthermore, hospitalization and mortality were associated with elevated levels of galectin-3^29,31^. This protein was shown to be involved in inflammation as well as the orchestration of the extracellular matrix and was identified to play a decisive role in cardiac remodeling^16^. Especially in inflammatory and fibrotic processes the level of galectin-3 was shown to be elevated^15,32^. Cardiac damage and inflammation lead to an increased expression of galectin-3 by activated macrophages, binding to a receptor on fibroblasts with consequent formation of cardiac fibrosis. Hence, over time interstitial fibrotic remodeling of the left ventricle leads to an impaired cardiac function^32^. Based on the underlying pathomechanisms of fibrosis and inflammation in hyperglycaemia-induced cardiac alteration, the biomarker galectin-3 may be a promising target in monitoring cardiac function especially in people with impaired glucose metabolism^33^, which was aimed to be investigated in the present study. Elevated galectin-3 levels were detected in acute and chronic as well as in end stage heart failure^30^. The protein was associated with prevalence of diabetes^33^ and was even found to play a role in the development of T2DM^34^. Further, the biomarker was related to the progression of nephropathy in diabetics. Galectin-3 was associated with cardiovascular events in diabetics with coronary artery disease, whereas NT-proBNP predicted events in patients without T2DM^35^. In accordance with the aforementioned study results, our study outlined that higher galectin-3 levels were associated with an increased risk for cardiovascular and all-cause mortality in individuals with T2DM. Thus, galectin-3 may be an important biomarker in T2DM and also be useful in patients with prediabetes.

Data regarding the validity and therefore the diagnostic value of galectin-3 compared to NT-proBNP are inconsistent in the literature, often due to small study populations in which this issue was investigated^36^. By this, an independent validity of galectin-3 was shown as well as an increase of the diagnostic value by the combination of galectin-3 with NT-proBNP but in contrast also, in other studies, a lack of an additional value by the aforementioned combination^27,36^. However, reflecting these results, studies with enough statistical power such as the GHS are required, and a more detailed look and distinction on the different entities of heart failure may identify possible issues in which galectin-3 may deliver further valuable information. With respect to heart failure with preserved ejection fraction, galectin-3 was shown to be of particular importance^37^. Since galectin-3 showed a strong association with impaired cardiac function especially in T2DM, the present data indicated that NT-proBNP, which additionally showed an association in prediabetes and euglycaemia, may have a higher prognostic value for diagnosing heart failure and evaluating the risk for mortality. In contrast to NT-proBNP, nevertheless, galectin-3 presented a prognostic value for systolic and diastolic dysfunction in T2DM. Hence, galectin-3 in general seems to be inferior to the established biomarker NT-proBNP in assessing and monitoring heart failure. According to the present data, however, galectin-3 may indeed deliver valuable additional information about the recent and future cardiac condition in individuals with T2DM. Of interest, the findings of the present study indicate a strong influence of cardiovascular risk factors on the prospective association between galectin-3 and diastolic dysfunction in T2DM, whereas comorbidities and heart failure medication intake seem to have a lower impact than CVRF. In line, the prognostic value of galectin-3 for incident HFpEF was demonstrated in patients with increased risk for heart failure^29^. Further, galectin-3 was associated to the development and progression of hypertension complicated with diastolic dysfunction^38^ and was suggested as a biomarker for early cardiac remodeling in patients with hypertension^39^. In young obese people without known cardiovascular disease, galectin-3 was related with preclinical metabolic heart disease and recommended as a screening tool for individuals with elevated risk for progression to obesity-related heart failure with preserved ejection fraction^40^. In glucose and lipid metabolism disorders, galectin-3 was demonstrated to promote cardiac remodeling by mediating cardiomyocyte fibrosis, apoptosis, and hypertrophy^41^. Furthermore, galectin-3 was shown to mediate autophagy and dysfunction of endothelial progenitor cells induced by cigarette smoke extract, suggesting galectin-3 to be involved in the detrimental effects of smoking on the cardiovascular system by comprising endothelial progenitor cells which have the potential to repair damaged blood vessels and promote angiogenesis^42^. Hence, galectin-3 seems to be crucially involved in the deleterious mechanisms of all cardiovascular risk factors. The present results on the diagnostic value of galectin-3 regarding cardiac function in euglycaemia, prediabetes and T2DM contribute to these pivotal findings.

Still, the role of galcetin-3 in diagnosing or monitoring cardiac function and its possible value in clinical practice remains contradictory. On the one hand, studies on heart failure revealed galectin-3 to correlate with the prevalence of diabetes mellitus and the metabolic syndrome^43^. Further, galectin-3 was identified as a regulatory factor in metabolic mechanisms like hyperglycaemia, lipogenesis as well as obesity induced inflammation and itself represents a risk factor for the development of heart failure and diabetes mellitus^44^. On the other hand, galectin-3 wields multivariable and to some extent contrary functions: galectin-3 deficient mice were associated with less atherosclerosis, inflammation and diabetes mellitus after infusion of streptozotocin. In contrast, these mice developed insulin resistance and glomerulopathy more rapidly and revealed higher concentrations of advanced glycation end products^45^. In inflammation, galectin-3 was shown to act inhibiting or accelerating depending on the stage of the inflammatory process: in acute inflammation galectin-3 acted protective, whereas in chronic inflammation it was shown to advance fibrosis and scarring. Regarding hyperglycaemia, high levels of galectin-3 were associated with lower levels of HbA1c^46^. On the other hand, galectin-3 is involved in numerous disease mechanisms, which play an important role in cardiac alteration due to diabetes mellitus. In this regard, galectin-3 acts as a receptor for advanced glycation end products as well as advanced lipoxidation end products and hence has an impact on inflammation and fibrosis^47^. This supports the results of the present study, in which galectin-3 correlated with an impaired systolic and diastolic function in individuals with T2DM and, regarding the predictive value for these special disease patterns, was superior to NT-proBNP.

According to the findings of the present study, T2DM might represent a possible circumstance in which galectin-3 may be a beneficial additional biomarker to NT-proBNP in order to monitor and predict the patients’ cardiac function.

### Strengths and limitations

The strength of the present study is based on the highly standardized investigation of a large population with more than 15,000 participants. The extrapolation of the results to other ethnicities has to be done with caution, since the study was performed in Mid-Western Germany and included mainly Caucasian people. Also, an extrapolation to cohorts with varying age ranges has to be done carefully. The present promising results have to be confirmed in future studies which need to further assess the role of galectin-3 in heart failure especially regarding individuals with impaired glucose metabolism. Hereby, additional echocardiographic parameters of cardiac function (like strain or left atrial size and function) as well as clinical variables (like incidental heart failure) should be involved, which were not addressed in the present study. However, it is striking that galectin-3 was predicting systolic and diastolic dysfunction in individuals with T2DM in a five-year period and in the latter was superior to NT-proBNP. According to these results, galectin-3 might be a promising additional biomarker for monitoring and prediction of cardiac function in T2DM.

## Conclusion

The present study represents a comprehensive analysis regarding the value of galectin-3 in detecting and predicting an impaired cardiac function in individuals with T2DM. In a large population-based cohort, galectin-3 was superior to NT-proBNP in predicting cardiac function in a five-years period in subjects with T2DM. Although inferior to NT-proBNP in the prediction of survival, galectin-3 may represent a valuable tool to deliver additional information in the monitoring and prediction of cardiac function in the high-risk group of patients with T2DM.

## Supplementary Information

Supplementary Information.

## Abbreviations

ATC code: Anatomical Therapeutic Chemical classification code of medication
BNP: Brain natriuretic peptide
CI: Confidence interval
CRP: C-reactive protein
CVD: Cardiovascular disease
CVRF: Cardiovascular risk factor(s)
EF: Ejection fraction
eGFR: Estimated glomerular filtration rate
FH of MI/stroke: Family history of myocardial infarction or stroke
GHS: Gutenberg Health Study
HFpEF: Heart failure with preserved ejection fraction
HFrEF: Heart failure with restricted ejection fraction
HR: Hazard ratio
SD: Standard deviation
T2DM: Type 2 diabetes mellitus
