# Supplementary material for: Galectin-3 for prediction of cardiac function compared to NT-proBNP in individuals with prediabetes and type 2 diabetes mellitus
Source: Sci Rep. 2021 Sep 24;11:19012. doi: 10.1038/s41598-021-98227-x (PMC8463561; doi:10.1038/s41598-021-98227-x)
Supplement: Supplementary file 1 — Supplementary Information. [file 41598_2021_98227_MOESM1_ESM.docx]

**SupplementaL Appendix**

**Galectin-3 for prediction of cardiac function compared to NT-proBNP in individuals with prediabetes and type 2 diabetes mellitus**

Volker H. Schmitt, MD^1,2^, Jürgen H. Prochaska, MD^3,4,2^, Annegret S. Föll, MD^3^, Andreas Schulz, PhD^3,4^, Karsten Keller, MD^1,4,5^, Omar, Hahad, PhD^1,2^, Thomas Koeck, PhD^2,4^, Sven-Oliver Tröbs, MD^1,2,3^, Steffen Rapp, PhD^3,4^, Manfred Beutel, MD^6^, Norbert Pfeiffer, MD^7^, Konstantin Strauch, PhD^8^, Karl J. Lackner, MD^9,2^, Thomas Münzel, MD^1,2,4*#^, Philipp S. Wild, MD, MSc^3,4,2*^

^1^Department of Cardiology, Cardiology I, University Medical Center, Johannes Gutenberg University Mainz, Langenbeckstr. 1, 55131 Mainz, Germany; ^2^German Center for Cardiovascular Research (DZHK), Partner Site Rhine-Main, Mainz, Germany; ^3^Preventive Cardiology and Preventive Medicine, Department of Cardiology, University Medical Center, Johannes Gutenberg University Mainz, Langenbeckstr. 1, 55131 Mainz, Germany; ^4^Center for Thrombosis and Hemostasis (CTH), University Medical Center, Johannes Gutenberg University Mainz, Langenbeckstr. 1, 55131 Mainz, Germany; ^5^Medical Clinic VII, Department of Sports Medicine, University Hospital Heidelberg, Im Neuenheimer Feld 410, 69120 Heidelberg, Germany; ^6^Department of Psychosomatic Medicine and Psychotherapy, University Medical Center, Johannes Gutenberg University Mainz, Langenbeckstr. 1, 55131 Mainz, Germany; ^7^Department of Ophthalmology, University Medical Center, Johannes Gutenberg University Mainz, Langenbeckstr. 1, 55131 Mainz, Germany; ^8^Institute of Medical Biostatistics, Epidemiology and Informatics (IMBEI), University Medical Center, Johannes Gutenberg University Mainz, Obere Zahlbacher Str. 69, 55131 Mainz, Germany; ^9^Institute of Clinical Chemistry and Laboratory Medicine, University Medical Center, Johannes Gutenberg University Mainz, Langenbeckstr. 1, 55131 Mainz, Germany

*both authors contributed equally and share senior authorship

^#^corresponding author

**SUPPLEMENTAL FIGURES**

**Supplemental Figure 1**

**Supplemental Figure legend 1**

Title: Distribution of people with euglycaemia, prediabetes and T2DM according to Galectin-3 tertiles.

Caption: With raising Galectin-3 levels the portion of euglycaemic people decreased while the amount of subjects with prediabetes and T2DM increased. In detail, in the first Galectin-3 tertile, 73% of the subject were euglycaemic, 22% prediabetic and 5% had T2DM. The second tertile consisted of 64% euglycaemics, 28% prediabetics and 8% diabetics. In the third quartile, 53% of subjects were euglycaemic whereas 33% had prediabetes and 14% had T2DM.

**SUPPLEMENTAL TABLES**

**Supplemental Table 1. Cross-sectional association between NT-proBNP and cardiac function in prediabetes and type 2 diabetes mellitus.**

|  | **Model 1: age, sex** | | **Model 2: add. traditional CVRF** | | **Model 3: add. traditional CVRF, comorbidities, heart failure medication** | |
| --- | --- | --- | --- | --- | --- | --- |
|  | **Estimate (95%CI)** | **P-value** | **Estimate (95%CI)** | **P-value** | **Estimate (95%CI)** | **P-value** |
| **Systolic function** | |  |  |  |  |  |
| EF in prediabetes vs. euglycaemia | -0.381  (-0.609; -0.153) | 0.0011 | -0.252  (-0.484; -0.0194) | 0.034 | -0.198  (-0.431; 0.0353) | 0.096 |
| EF in diabetes vs. euglycaemia | -0.446  (-0.818; -0.0751) | 0.018 | -0.155  (-0.542; 0.232) | 0.43 | -0.0344  (-0.427; 0.358) | 0.86 |
| EF ~ NT-proBNP [SD] in euglycaemia | -0.659  (-0.796; -0.521) | < 0.0001 | -0.671  (-0.809; -0.534) | < 0.0001 | -0.584  (-0.724; -0.444) | < 0.0001 |
| EF ~ NT-proBNP [SD] in prediabetes | -1.30  (-1.49; -1.11) | < 0.0001 | -1.35  (-1.54; -1.15) | < 0.0001 | -1.14  (-1.34; -0.948) | < 0.0001 |
| EF ~ NT-proBNP [SD] in diabetes | -1.70  (-1.98; -1.42) | < 0.0001 | -1.70  (-1.98; -1.42) | < 0.0001 | -1.43  (-1.72; -1.14) | < 0.0001 |
| **Diastolic function** | |  |  |  |  |  |
| log (E/E´) in prediabetes vs. euglycaemia | 0.0401  (0.0293; 0.0510) | < 0.0001 | 0.0166  (0.00585; 0.0274) | 0.0025 | 0.0171  (0.00624; 0.028) | 0.002 |
| log (E/E´) in diabetes vs. euglycaemia | 0.127  (0.109; 0.144) | < 0.0001 | 0.0643  (0.0464; 0.0823) | < 0.0001 | 0.0587  (0.0404; 0.077) | < 0.0001 |
| log (E/E´) ~ NT-proBNP [SD] in euglycaemia | 0.0152  (0.00869; 0.0218) | < 0.0001 | 0.0164  (0.00998; 0.0228) | < 0.0001 | 0.0147  (0.00813; 0.0212) | < 0.0001 |
| log (E/E´) ~ NT-proBNP [SD] in prediabetes | 0.0151  (0.00605; 0.0242) | 0.0011 | 0.0176  (0.00874; 0.0265) | 0.0001 | 0.0166  (0.00748; 0.0258) | 0.00036 |
| log (E/E´) ~ NT-proBNP [SD] in diabetes | 0.0475  (0.0342; 0.0607) | < 0.0001 | 0.0455  (0.0325; 0.0585) | < 0.0001 | 0.0435  (0.0303; 0.0568) | < 0.0001 |
| Multiple linear regression models for investigation of the association between NT-proBNP (increase per standard deviation respectively 5 ng/ml) and EF as well as log(E/E´) in prediabetes and diabetes compared to euglycaemia within the baseline data. Model 1 adjusted for sex and age. Model 2 adjusted for sex, age, hypertension, dyslipidaemia, obesity, smoking, FH of MI/Stroke. Model 3 adjusted for sex, age, hypertension, dyslipidaemia, obesity, smoking, FH of MI/Stroke, atrial fibrillation, chronic kidney disease, chronic liver disease, congestive heart failure, coronary artery disease, myocardial infarction, peripheral artery disease, stroke, venous thromboembolism, heart failure medication intake. CVRF: cardiovascular risk factors; SD: standard deviation. | | | | | | |

**Supplemental Table 2. Prospective association between NT-proBNP and cardiac function in prediabetes and type 2 diabetes mellitus.**

|  | **Model 1: age, sex** | | **Model 2: add. traditional CVRF** | | **Model 3: add. traditional CVRF, comorbidities, heart failure medication** | |
| --- | --- | --- | --- | --- | --- | --- |
|  | **Estimate (95%CI)** | **P-value** | **Estimate (95%CI)** | **P-value** | **Estimate (95%CI)** | **P-value** |
| **Systolic function** | |  |  |  |  |  |
| EF in prediabetes vs. euglycaemia | 0.0715  (-0.238; 0.381) | 0.65 | 0.287  (-0.0272; 0.601) | 0.073 | 0.305  (-0.0118; 0.622) | 0.059 |
| EF in diabetes vs. euglycaemia | -1.75  (-2.25; -1.25) | < 0.0001 | -1.30  (-1.82; -0.778) | < 0.0001 | -1.23  (-1.76; -0.698) | < 0.0001 |
| EF ~ NT-proBNP [SD] in euglycaemia | 0.123  (-0.0472; 0.294) | 0.16 | 0.0989  (-0.0717; 0.269) | 0.26 | 0.137  (-0.0363; 0.311) | 0.12 |
| EF ~ NT-proBNP [SD] in prediabetes | -0.489  (-0.747; -0.231) | 0.00021 | -0.531  (-0.789; -0.273) | < 0.0001 | -0.385  (-0.649; -0.12) | 0.0043 |
| EF ~ NT-proBNP [SD] in diabetes | -1.14  (-1.54; -0.746) | < 0.0001 | -1.23  (-1.63; -0.829) | < 0.0001 | -1.07  (-1.48; -0.668) | < 0.0001 |
| **Diastolic function** | |  |  |  |  |  |
| log (E/E´) in prediabetes vs. euglycaemia | 0.0209  (0.00773; 0.0341) | 0.0019 | 0.0114  (-0.00192; 0.0248) | 0.093 | 0.0118  (-0.00173; 0.0253) | 0.087 |
| log (E/E´) in diabetes vs. euglycaemia | 0.0614  (0.0399; 0.0829) | < 0.0001 | 0.0387  (0.0166; 0.0608) | 0.0006 | 0.039  (0.0165; 0.0616) | 0.00071 |
| log (E/E´) ~ NT-proBNP [SD] in euglycaemia | -0.0105  (-0.0178; -0.00329) | 0.0044 | -0.00990  (-0.0171; -0.00266) | 0.0074 | -0.0109  (-0.0183; -0.00353) | 0.0038 |
| log (E/E´) ~ NT-proBNP [SD] in prediabetes | -0.00441  (-0.0154; 0.00653) | 0.43 | -0.00339  (-0.0143; 0.00752) | 0.54 | -0.00574  (-0.0169; 0.00547) | 0.32 |
| log (E/E´) ~ NT-proBNP [SD] in diabetes | 0.00515  (-0.0117; 0.0220) | 0.55 | 0.00832  (-0.00852; 0.0252) | 0.33 | 0.00507  (-0.0122; 0.0223) | 0.56 |
| Prospective analyses using multiple linear regression models for the assessment of the association between NT-proBNP (increase per standard deviation respectively 5 ng/ml) and EF as well as log(E/E´) after 5 years in prediabetes and diabetes compared to euglycaemia. Individuals with impaired systolic or diastolic function were excluded from the follow-up investigation. Model 1 adjusted for sex and age. Model 2 adjusted for sex, age, hypertension, dyslipidaemia, obesity, smoking, FH of MI/Stroke. Model 3 adjusted for sex, age, hypertension, dyslipidaemia, obesity, smoking, FH of MI/Stroke, atrial fibrillation, chronic kidney disease, chronic liver disease, congestive heart failure, coronary artery disease, myocardial infarction, peripheral artery disease, stroke, venous thromboembolism, heart failure medication intake. CVRF: cardiovascular risk factors; SD: standard deviation. | | | | | | |

**Supplemental Table 3. Association between NT-proBNP and cardiovascular as well as all-cause mortality in prediabetes and type 2 diabetes mellitus.**

|  | **Model 1: age, sex** | | | | | **Model 2: age, sex, traditional CVRF** | | | | **Model 3: age, sex, traditional CVRF, EF, log(E/E’)** | | | | **Model 4: age, sex, traditional CVRF, cancer, eGFR** | | |
| --- | --- | --- | --- | --- | --- | --- | --- | --- | --- | --- | --- | --- | --- | --- | --- | --- |
|  | **Hazard ratio (95%CI)** | | | **P-value** | | **Hazard ratio (95%CI)** | | **P-value** | | **Hazard ratio (95%CI)** | | **P-value** | | **Hazard ratio (95%CI)** | | **P-value** |
| **All-cause mortality** | | |  | |  | |  | |  | |  | |  | |  | |
| log(NT-proBNP) [SD] in euglycaemia | 1.579  (1.429; 1.745) | | | < 0.0001 | | 1.597  (1.443; 1.767) | | < 0.0001 | | 1.519  (1.371; 1.683) | | < 0.0001 | | 1.587  (1.433; 1.758) | | <0.0001 |
| log(NT-proBNP) [SD] in prediabetes | 1.551  (1.392; 1.727) | | | < 0.0001 | | 1.543  (1.387; 1.716) | | < 0.0001 | | 1.447  (1.296; 1.616) | | < 0.0001 | | 1.563  (1.404; 1.740) | | <0.0001 |
| log(NT-proBNP) [SD] in diabetes | 2.187  (1.992; 2.401) | | | < 0.0001 | | 2.047  (1.860; 2.252) | | < 0.0001 | | 1.889  (1.707; 2.090) | | < 0.0001 | | 2.088  (1.892; 2.305) | | <0.0001 |
| **Cardiovascular mortality** | |  | | |  | |  | |  | |  | |  | | | |
| log(NT-proBNP) [SD] in euglycaemia | 1.862  (1.397; 2.483) | | | < 0.0001 | | 1.924  (1.437; 2.577) | | < 0.0001 | | 1.726  (1.273; 2.342) | | 0.00045 | | 1.846  (1.369; 2.488) | | <0.0001 |
| log(NT-proBNP) [SD] in prediabetes | 1.688  (1.193; 2.390) | | | 0.0031 | | 1.634  (1.168; 2.286) | | 0.0041 | | 1.494  (1.060; 2.105) | | 0.022 | | 1.631  (1.165; 2.282) | | 0.0043 |
| log(NT-proBNP) [SD] in diabetes | 3.033  (2.459; 3.742) | | | < 0.0001 | | 2.751  (2.202; 3.438) | | < 0.0001 | | 2.403  (1.921; 3.007) | | < 0.0001 | | 2.681  (2.135; 3.367) | | <0.0001 |
| Cox regression models for investigation of the association between NT-proBNP (increase per standard deviation respectively 5 ng/ml) and cardiovascular as well as all-cause mortality in prediabetes and diabetes compared to euglycaemia. Model 1 adjusted for sex and age; n = 14,168; CV mortality events = 108; all-cause mortality events = 811. Model 2 adjusted for sex, age, hypertension, dyslipidaemia, obesity, smoking, FH of MI/stroke. n = 14,118; CV mortality events = 107; all-cause mortality events = 804. Model 3 adjusted for sex, age, hypertension, dyslipidaemia, obesity, smoking, FH of MI/stroke, EF, log(E/E'). n = 13,954; CV mortality events = 106; all-cause mortality events = 793. Model 4 adjusted for sex, age, hypertension, dyslipidaemia, obesity, smoking, FH of MI/stroke, cancer, eGFR. n = 14,106; CV mortality events = 107; all-cause mortality events = 803. EF: ejection fraction; CVRF: cardiovascular risk factors; FH of MI/stroke: Family history of myocardial infarction or stroke; SD: standard deviation. | | | | | | | | | | | | | | | | |
